# Extracellular Vesicle-Dependent Communication Between Mesenchymal Stromal Cells and Immune Effector Cells

**DOI:** 10.3389/fcell.2020.596079

**Published:** 2020-11-06

**Authors:** Riccardo Bazzoni, Paul Takam Kamga, Ilaria Tanasi, Mauro Krampera

**Affiliations:** ^1^Stem Cell Research Laboratory, Section of Hematology, Department of Medicine, University of Verona, Verona, Italy; ^2^EA4340-BCOH, Biomarker in Cancerology and Onco-Haematology, UVSQ, Université Paris Saclay, Boulogne-Billancourt, France

**Keywords:** extracellular vesicles, exosomes, microvesicles, mesenchymal stromal cells, immune effector cells, immunomodulation

## Abstract

Mesenchymal stem/stromal cells (MSCs) are multipotent cells residing in the stromal tissues of the body and capable of promoting tissue repair and attenuating inflammatory processes through their immunomodulatory properties. Preclinical and clinical observations revealed that not only direct intercellular communication mediates MSC properties; in fact, a pivotal role is also played by the release of soluble and bioactive factors, such as cytokines, growth factor and extracellular vesicles (EVs). EVs are membrane-coated vesicles containing a large variety of bioactive molecules, including lipids, proteins, and nucleic acids, such as RNA. EVs release their contents into target cells, thus influencing cell fate through the control of intracellular processes. In addition, MSC-derived EVs can mediate modulatory effects toward different effector cells belonging to both innate and adaptive immunity. In this review, we will discuss the literature data concerning MSC-derived EVs, including the current standardized methods for their isolation and characterization, the mechanisms supporting their immunoregulatory properties, and their potential clinical application as alternative to MSC-based therapy for inflammatory reactions, such as graft-versus-host disease (GvHD).

## Introduction

Mesenchymal stromal cells (MSCs) are multipotent stem cells of mesodermal origin described in bone marrow (BM) for the first time by Alexander Friedenstein in 1966 (Friedenstein et al., 1966). Over the last decades, MSCs were also identified in a large number of tissues, including fat, umbilical cord, amniotic fluid, placenta, skin, dental pulp, and many others (Riekstina et al., 2008; Marquez-Curtis et al., 2015; Camilleri et al., 2016; Ventura Ferreira et al., 2018; Caseiro et al., 2019; Fukutake et al., 2019). In 2006, the International Society for Cellular Therapy (ISCT) established the minimal criteria to define human MSCs, i.e., (i) plastic-adherence when maintained in standard culture conditions; (ii) surface expression of CD105, CD73 and CD90 antigens while lacking CD45, CD34, CD14 or CD11b, CD79α or CD19, and HLA-DR molecules; (iii) *in vitro* differentiation into three mesodermal lineages (osteoblasts, adipocytes, and chondrocytes) (Dominici et al., 2006). MSCs boosted a great interest in the field of regenerative medicine and tissue engineering thanks to their ability to promote tissue regeneration and to modulate immune response (de Mayo et al., 2017; Petri et al., 2017; Pokrywczynska et al., 2019). Indeed, MSCs possess broad immunomodulatory properties affecting immune effector cells of both innate and adaptive responses (Krampera, 2011). For example, MSCs are capable of stimulating cytokine release and proliferation of innate lymphoid cells (van Hoeven et al., 2018), affecting dendritic cell (DC) maturation and activation (Zhang et al., 2004), suppressing natural killer (NK) cell activity and proliferation (Spaggiari et al., 2008), supporting the expansion of myeloid-derived suppressor cells (MDSCs) (Yen et al., 2013), and regulating B cell proliferation and activation (Fan et al., 2016) as well as T cell activity, balance between T helper (Th)1 and Th2 lymphocytes and expansion of T regulatory (Treg) cells (Haddad and Saldanha-Araujo, 2014; Gao et al., 2016). The ability of MSCs to modulate the immune response is well documented by several preclinical and clinical studies in a wide range of inflammatory and autoimmune diseases, such as Crohn’s disease (Forbes, 2017), rheumatoid arthritis (Ansboro et al., 2017), diabetes (Cho et al., 2018), graft-versus-host disease (GvHD) (Le Blanc et al., 2008), sepsis (Hall et al., 2013), cardiovascular diseases (Bagno et al., 2018), allergic airway inflammation (Takeda et al., 2018), and many others. Initially, the biological activity of MSC was ascribed to their ability to home within the injury site; however, only a small fraction of MSCs is capable of reaching the damaged tissues after systemic administration (Kraitchman et al., 2005; Yukawa et al., 2012; Scarfe et al., 2018), while the majority of them are rapidly cleared through phenomena of efferocytosis, thus polarizing macrophages toward an inhibitory phenotype (Galleu et al., 2017). In addition, MSCs may act at paracrine level through the release of bioactive factors, including transforming growth factor β (TGF-β), hepatocyte growth factor, prostaglandin E2 (PGE2), interleukin (IL)-10 and IL-6, human leukocyte antigen G (HLA-G), indoleamine-2,3-dioxygenase (IDO), nitric oxide (NO), and other mediators (Sato et al., 2006; Ryan et al., 2007; Németh et al., 2009; Bouffi et al., 2010; Du et al., 2016; Wang et al., 2018; Liu et al., 2019; Lu et al., 2019; Pittenger et al., 2019). In the last years, membrane-bound particles, known as extracellular vesicles (EVs), have been recognized as an important MSC paracrine factor in addition to soluble factors (Chen et al., 2016; Bier et al., 2018). EVs represent a very effective, physiological intercellular communication, even at low molecule concentrations at which soluble factors could be rapidly inactivated. Strong experimental evidence shows that MSC-EVs are capable of recapitulating the immunomodulation of their parental cells (Rani et al., 2015; Seo et al., 2019). Therefore, in this review we will provide an overview of the literature data supporting the MSC-EV-dependent communication between MSCs and immune effector cells (IECs).

## Characterization of EVs

EVs consist of a phospholipid bilayer envelope acting as molecular shuttle for various molecules, such as proteins, different types of nucleic acids, lipids and active metabolites (Lai et al., 2016; Yuan et al., 2017; Yang et al., 2018; Shojaati et al., 2019). Historically, EVs are classified into three main groups according to their biogenesis and size: (i) exosomes, (ii) microvesicles and (iii) apoptotic bodies. Exosomes (diameter range 50–100 nm) represent the smallest EV fraction deriving from the fusion of intracellular endosomes with plasma membrane, followed by their release into the extracellular space (Stephen et al., 2016). The production of exosomes is generally constitutive, although it can increase upon cell stimulation (Fierabracci et al., 2015). Microvesicles (MVs; diameter range 100–1,000 nm) are generated by cytoplasmic membrane budding in response to several stimuli resulting in cytosolic Ca^2+^ increment and disassembly of the cytoskeleton (Ratajczak et al., 2006). Apoptotic bodies (diameter range 1–5 μm) are characterized by irregular shapes and heterogenous sizes (Caruso and Poon, 2018). Apoptotic bodies are functionally different, as they are released during apoptosis and contain mainly cellular debris, such as micronuclei, chromatin remnants and cytosol portions (Battistelli and Falcieri, 2020). As several studies were performed with different separation approaches and cellular sources of EVs, it is still not possible to propose a specific classification of different EV subtypes as well as specific markers and biogenesis processes (Gould and Raposo, 2013; Cocucci and Meldolesi, 2015). Consequently, the Minimal Information of Studies of Extracellular Vesicles 2018 (MISEV2018) suggests to use the generic terms “small/medium/large EVs,” according to their size or density, instead of the classical “exosomes,” “microvesicles,” and “apoptotic bodies” terms (Théry et al., 2018). According to MISEV2018, to confirm the nature of EVs and the degree of purity of EV preparation, the scientific community has encouraged to evaluate the presence of at least one of transmembrane or GPI-anchored proteins associated to plasma membrane and/or endosomes (for example tetraspanins, integrins, and MHC class I) and cytosolic proteins recovered in EVs (for example lipid or membrane protein-binding ability like ESCRT-I/II/III and ALIX or promiscuous proteins like HSP70 or cytoskeleton proteins like actin and tubulin) and major components of non-EV co-isolated structures (for example lipoproteins, protein/nucleic acid aggregates, and ribosomal proteins) (Théry et al., 2018). Additionally, for studies focused on one or more EV subtypes is recommended to assess the presence of transmembrane, lipid-bound and soluble proteins associated to other intracellular compartments than plasma membrane/endosomes, including lamin A/C, cytochrome C, calnexin, and ATG9A, whereas for the evaluation of EV functional activities, the identification of functional soluble factor in EVs like cytokines, growth factors, adhesion and extracellular matrix proteins is required (Théry et al., 2018).

The communication system based on EVs is highly conserved among the three different animal reigns, thus suggesting how EVs are crucial for intercellular communication (Deatherage and Cookson, 2012; Gill et al., 2019). EVs contribute to cell-to-cell communication via direct contact with target cells through a ligand–receptor interaction. In particular, EVs can transfer information to target cells either without delivering their content or acting like biological shuttles that release their cargo into acceptor cells. A classic example of EV contribution to intercellular communication without deliver their content resides in those vesicles that harbor MHC molecules on their surface, thus activating T cell receptors on T cells (Raposo et al., 1996; Martin et al., 2014). Concerning the delivery of EV content, EVs can be taken up by target cells through several mechanisms, including clathrin-mediated endocytosis, caveolin-dependent endocytosis, macropinocytosis, phagocytosis, lipid rafts, and cell surface membrane fusion (Feng et al., 2010; Montecalvo et al., 2012; Svensson et al., 2013; Tian et al., 2014; Costa Verdera et al., 2017; Rai and Johnson, 2019). Although numerous receptors/ligands are implicated into EV uptake including tetraspanins, integrins, immunoglobulins, lectins, and proteoglycans (Morelli et al., 2004; Hao et al., 2007; Barrès et al., 2010; Christianson et al., 2013), to date it is still debated whether EV uptake is a cell-type specific process or not. Indeed, some studies suggest that EVs from different sources can be taken up by every cell type (Costa Verdera et al., 2017; Horibe et al., 2018), whereas others report that only a particular combination of EV and target cells (and thus the right association between receptors and ligands) allow the EV uptake by acceptors cells (Fitzner et al., 2011; Zech et al., 2012; Chivet et al., 2014; Di Trapani et al., 2016). Finally, recent evidence suggested that nanotubes could synergistically act with EVs in intercellular communication, as microsized particles could be transferred into target cells via nanotubes (Ware et al., 2015; Nawaz and Fatima, 2017).

As EVs reflect the characteristics of their cells of origin both at molecular and functional level, EVs have emerged as a novel potential therapeutic approach due to their ability to influence various biological processes, including immune response, cell proliferation, tissue regeneration, cell invasiveness, tubule formation, angiogenesis, synapsis plasticity, and many others (Zaborowski et al., 2015; Silva et al., 2017; Prada et al., 2018; Lee et al., 2019; Mou et al., 2019).

## MSC-EVs and Immunomodulation

**FIGURE 1:**
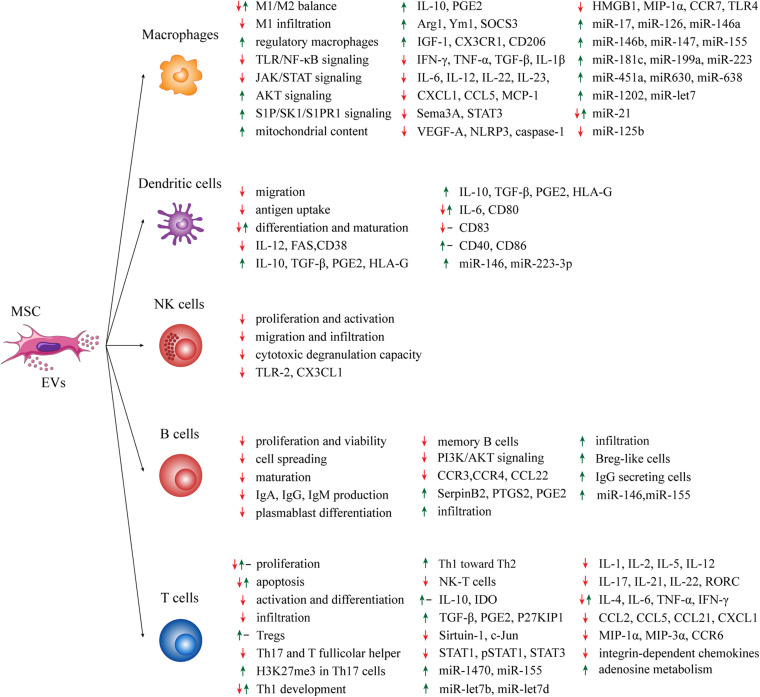
Summary of the effects of MSC-EVs on immune effector cells. Green arrows: positive effect; red arrows: negative effect; black dashes: no effect.

MSC-EVs play a pivotal role in mediating the paracrine effects of MSCs on immune system. Generally, MSC-EVs may promote an immunosuppressive response through the induction of immature DCs, the polarization of macrophages toward M2-like phenotype, the inhibition of immunoglobulin (Ig) release, the expansion of Tregs and the secretion of anti-inflammatory cytokines (Budoni et al., 2013; Burrello et al., 2016; Favaro et al., 2016; Balbi et al., 2017; Du et al., 2018). However, MSC-EVs should be considered in the whole contest of MSC secretome, because in some experimental settings the immunomodulation mediated by MSC-EVs can only poorly recapitulate the immune properties of their parental cells (Conforti et al., 2014; Gouveia de Andrade et al., 2015; Ma et al., 2019). In the next sections we will try to give a comprehensive overview of the effects of MSC-EVs on the innate (macrophages, DCs and NK cells) and adaptive (B and T cells) immune system. As the studies here reported employed different EV subtypes obtained from several MSC sources (BM, umbilical cord, adipose tissue, fetal liver) of different animal species (human, mouse and rat) with several isolation methods, we will refer to them with the generic term “MSC-EVs.” The immunomodulatory effects of MSC-EVs on innate and adaptive immune system are summarized in Figure 1.

### MSC-EVs and Innate Immune System

#### Macrophages

Macrophages are mononuclear phagocytes with important roles in physiological conditions and in first-line immune response (Zhang and Wang, 2014). Macrophages are extremely plastic cells, with the capability of differentiating into two activated subtypes, i.e., M1 and M2. M1 macrophages are classical activated cells secreting large amount of pro-inflammatory factors, such as TNF-α, IL-1β and reactive oxygen species. On the other hand, M2 macrophages are alternatively activated and anti-inflammatory cells producing IL-10 and trophic factors (Shapouri-Moghaddam et al., 2018). Recent data support a contribution of MSC-EVs in modulating the M1/M2 balance, although the precise mechanism remains unclear. For instance, MSC-EVs may hamper the activation of pro-inflammatory M1 macrophages in favor of pro-resolving M2 macrophages that parallel with VEGF-A, IFN-γ, IL-12, and TNF-α reduction as well as IL-10 upregulation (Balbi et al., 2017; Cosenza et al., 2017; Cao et al., 2019). The modulation of several signaling pathways mediated by MSC-EVs may be responsible for this effect. For instance, the inhibition of JAK/STAT signaling was confirmed by many studies, resulting in Arg1 increment and inflammation reduction (Zhao et al., 2018; Cao et al., 2019). The activation of S1P/SK1/S1PR1 signaling by MSC-EVs promotes M2 differentiation through the downregulation of NF-κB-p65 and TGF-β1 expression in macrophages, thus restoring cardiac activity after myocardial infarction (Deng et al., 2019). Furthermore, lipopolysaccharide (LPS)-primed MSC-EVs support M2 macrophage polarization, by interfering with LPS−dependent NF−κB signaling, and partly activate the AKT1/AKT2 signaling pathway, by attenuating the post-infarction inflammation and cardiomyocyte apoptosis (Xu et al., 2019). An interesting mechanism by which MSC-EVs exert their anti-inflammatory function is the M2 polarization through MSC-EV-mediated mitochondrial transfer that is dependent on macrophage oxidative phosphorylation (Morrison et al., 2017). In parallel, in response to oxidative stress, MSCs outsource mitochondria depolarized by MVs, thus enhancing macrophage bioenergetics (Phinney et al., 2015) and therefore their pro-inflammatory features (Tavakoli et al., 2013). Moreover, MSCs-EVs may trigger the anti-inflammatory phenotype and pro-resolving properties of mature, human regulatory macrophages, a subclass of M2 macrophages characterized by modest IL-22 and IL-23 production and PGE2 hyper-expression, thus leading to reduction of Th17 response (Hyvärinen et al., 2018). MSC-EVs can also reduce chemokine expression (CXCL1 and CCL5) that are necessary for inflammatory response by macrophages (Zou et al., 2014; Willis et al., 2017). Interestingly, MSC-EVs express CCR2 chemokine, bind and reduce the concentration of the free pro-inflammatory CCL2 ligand, and therefore prevent the activation and recruitment of M1 macrophages (Shen et al., 2016). MSC-EVs may also trigger the anti-inflammatory phenotype in hepatic macrophages through IGF-1 (Fiore et al., 2020).

Several miRNAs are involved into MSC-EV-mediated anti-inflammatory effects on M1/M2 balance. For instance, miR-223 overexpression in MSC-EVs may reprogram macrophages from M1 to M2 phenotype by targeting Sema3A and STAT3 (Wang et al., 2015; He et al., 2019). Under hypoxic condition, the enrichment of miR-223 determines the overexpression of miR-146b, miR-126 and miR-199a, which in turn upregulate the expression of Arg1 and Ym1 and promote the anti-inflammatory M2 state (Lo Sicco et al., 2017). Other miRNAs involved in M2 polarization are miR-155 and miR-21, whose downregulation results in the increase of SOCS3 and M2 molecules (IL-10, CD206 and arginase) as well as M1 marker reduction (CCR7, IL-1β, IL-6, and NO) (Henao Agudelo et al., 2017). IL-1β-primed MSC-EVs express high levels of miR-146a promoting M2 macrophage polarization more effectively than IL-1β-primed MSC, thus increasing survival of septic mice (Song et al., 2017). The comparative miRNA analysis of EVs isolated from either IFN-γ-primed or resting MSCs revealed that miR-150-5p, whose target genes is involved in acute-phase response and signaling in macrophages, is downregulated in IFN-γ-primed EVs. Nevertheless, no difference between primed and resting EVs has been observed in promoting macrophage differentiation toward M2 phenotype (Marinaro et al., 2019). The enrichment of miR-let7 within MSC-EVs may favor M2 polarization and suppress macrophage infiltration through miR-let7/HMGA2/NF-κB pathway and miR-let7/IGF2BP1/PTEN pathway, respectively (Li et al., 2019). Moreover, LPS-primed MSC-EVs regulate the M1/M2 macrophage balance more efficiently than resting EVs, thanks to the expression of miR-let-7b, which inhibits TLR4/NF-κB/STAT3/AKT signaling pathway, thus hampering inflammation and enhancing diabetic cutaneous wounds healing (Ti et al., 2015). TLR/NF-κB signaling can be targeted by other miRNAs too. In a model of burn-induced inflammation, the administration of MSC-EVs overexpressing miR-181c reduced the number of macrophages (and neutrophils) potentially inhibiting TLR4 expression and its downstream target proteins NF-κB/P65 and p-65, thus preventing inflammation (Li et al., 2016). MSC-EV fraction is characterized by the enrichment in miR-451a, miR-1202, miR-630, and miR-638 and the reduced expression of miR-125b and miR-21. This miRNA profile may be responsible for targeting MYD88-dependent inflammatory nodes to suppress TLR/NF-κB signaling pathway and macrophage activation (Phinney et al., 2015). Additionally, Zhang et al. found *in vitro* that MSC-EVs induce monocytes to switch toward an anti-inflammatory M2-phenotype via MYD88-dependent TLR signaling pathway, resulting in a reduction of IL-1β, IL-6, IL-12, and TNF-α levels and higher IL-10 concentration, thus polarizing activated CD4^+^ T cells toward Treg subset (Zhang et al., 2013).

MSC-EVs also prevent M1-type macrophage infiltration in injury sites by lowering MCP-1, CCL5, HMGB1, and MIP-1α expression (Yu et al., 2016; Spinosa et al., 2018; Woo et al., 2020), probably through miR-147 expression (Spinosa et al., 2018). Interestingly, in a model of thioglycolate-induced peritonitis, treatment with MSC-EVs reduced macrophage infiltration in the peritoneal cavity by inducing a M2-like regulatory phenotype; this effect was partially associated to the upregulation of CX3CR1 in F4/80^+^/Ly6C^+^/CCR2^+^ macrophage subset (Henao Agudelo et al., 2017). Concerning the homing ability of MSC-EVs, Lankford et al. demonstrated in a model of damaged spinal cord that MSC-EVs can migrate only in the injury site and M2-type macrophage are the primary target of EVs (Lankford et al., 2018).

Finally, MSC-EVs mediate miR-17 transfer from parental cells to macrophages, thus suppressing NLRP3 inflammasome activation, and consequently caspase-1, IL-1β, and IL-6, by targeting TXNIP (Liu et al., 2018). The suppression of NLRP3, caspase-1, IL-1β, and IL-6 was also reported by other authors (Jiang et al., 2019). On the other hand, EVs isolated from LPS-primed periodontal ligament stem cells (characterized by MSC-like markers) may induce strong M1-type polarization in association with pro-inflammatory molecules (TNF-α and IL-6); this effect seems related to double-strand DNA on EV surface (Kang et al., 2018).

#### Dendritic Cells

DCs are innate professional antigen-presenting cells (APCs) acting as central regulators of the adaptive immune response. DCs can be found in either resting or active state. Resting DCs are immature APCs expressing low levels of costimulatory molecules (CD38, CD40, CD80, CD83, and CD86) and immunostimulatory cytokines conferring high capacity to capture antigens. DC activation and maturation depend on different stimuli deriving from bacteria, viruses and damaged tissue. Activated DCs are potent T cell response inducers showing low antigen capture activity and high expression of histocompatibility complex II (MHC class II), costimulatory signals, C-C chemokine receptor type 7 (CCR7) as well as immunostimulatory cytokines (Collin et al., 2013; Patente et al., 2019). EVs secreted by different types of MSCs exert immunosuppressive effects on DCs primarily by inhibiting their activation, eventually leading to the lack of T cell response triggering. For example, DCs from type 1-diabetic (T1D) patients treated with heterologous MSC-EVs acquired an immature phenotype, characterized by low expression of activation markers and higher production of IL-6, IL-10, TGF-β, and PGE2 (Favaro et al., 2016). Therefore, MSC-EV-treated DCs inhibit the inflammatory T cell response by decreasing Th17 subset and inducing Foxp3^+^ Tregs (Favaro et al., 2016). Similarly, MSC-EV treatment leads to anergic, IL-10-expressing, regulatory DCs that suppress Th1 and Th17 cell development, but without inducing Tregs (Shigemoto-Kuroda et al., 2017). Notably, MSC-EVs may enhance the release of TGF-β and IL-10 from CD11c^+^ DCs, thus inhibiting lymphocyte proliferation, without affecting the expression of MHC class II, CD86, CD83, and CD40 (Shahir et al., 2020). Upregulation of miR-146 expression in DCs is a possible mechanism by which MSC-EVs promote DC immature phenotype, leading to the downregulation of FAS expression and IL-12 production (Wu et al., 2017). Alternatively, EVs derived from renal, mesenchymal-like cancer stem cells impair dendritic differentiation and T cell activation by upregulating the expression of the anti-inflammatory molecule HLA-G (Grange et al., 2015). MSC-EVs may also prevent immature DCs from antigen uptake by blocking their maturation (Reis et al., 2018). As a consequence, MSC-EVs lower CD38, CD80, CD83, IL-6, and IL-12 expression, increase the production of the anti-inflammatory cytokine TGF-β and reduce DC ability to migrate toward CCL21, the CCR7-ligand, although DCs can still trigger allogeneic T cell proliferation *in vitro* (Reis et al., 2018). These MSC-EV-treated DCs resulted enriched of four microRNAs (miR-21-5p, miR-142-3p, miR-223-3p, and miR-126-3p) mediating well-known effects on DC maturation and functions (Reis et al., 2018). On the other hand, higher expression of costimulatory factors (CD40, CD80, and CD86), but not MHC class II, can be observed on the surface of murine immature DCs following MSC-EV treatment, thus suggesting that these EVs can mediate the DC maturation required for the induction of effector T-cell (Cho et al., 2019).

#### Natural Killer Cells

NK cells are lymphoid cells with a central role in the innate response to viral infections and cancer cells, but recent data suggest that NK cells can also modulate the adaptive immune response involving DCs and T cells, either directly or indirectly (Moretta et al., 2008; Chiossone et al., 2018). Despite a deep search in literature, only a few papers concerning the role of MSC-EVs on NK cell modulation have been found. EVs derived from MSCs. EVs prevent proliferation and IL-2-induced activation of both CD56-dim and CD56-bright NK cells, and suppressed their cytotoxic degranulation *in vitro* (Fan et al., 2018). In a rat model of experimental autoimmune uveitis (EAU), MSC-EV administration reduces CD161^+^ NK cell migration toward eye lesions, thus ameliorating EAU symptoms (Bai et al., 2017). The protective and anti-inflammatory effects exerted by MSC-EVs have been also observed in a rat model of renal ischemic reperfusion injury (IRI) and in a renal allografts MHC-disparate rat model, by decreasing both NK cells infiltration and chemokines associated with NK cell recruitment (TLR-2 and CX3CL1) (Koch et al., 2015; Zou et al., 2016). All these immunosuppressive effects seem to be mediated by the expression of TGF-β on the EV surface, which induces TGF-β/Smad downstream pathway (Fan et al., 2018). Other molecules contained in MSC-EVs and associated with anti-inflammatory effects on NK cells are IL-10 and HLA-G (Kordelas et al., 2014). Finally, TNF-α- and IFN-γ-primed MSC-EVs reduce NK cell proliferation more effectively than resting MSC-EVs (Di Trapani et al., 2016).

### MSC-EVs and Adaptive Immune System

#### B Cells

B cells are lymphoid cells involved in the humoral adaptive immunity through the secretion of antibodies and cytokines (Matsushita, 2019). Among the peripheral blood mononuclear cell (PBMC) subpopulations, B cells show the highest EV uptake (Di Trapani et al., 2016). MSC-EVs may induce in B cells the downregulation of 11 genes (including CCR3, CCR4, and CCL22) and the upregulation of 39 genes (including SerpinB2, PTGS2, and PGE2) involved in immune regulation (Khare et al., 2018). MSC-mediated inhibition of B cell proliferation is more evident following inflammatory priming (Di Trapani et al., 2016). Inflammatory priming induces the increase of miR-155 and miR-146 levels within MSC-EVs (Di Trapani et al., 2016). In particular, MSC-EVs induce the downregulation of PI3K/AKT signaling pathway components in B cells, inhibit B cell spreading, and reduce B cell viability via miR-155-5p (Adamo et al., 2019).

Another effect of MSC-EVs on B cells is preventing Ig secretion. MSC-EVs exert a dose-dependent inhibition of IgM, IgG, and IgA production coupled with suppression of B cell proliferation and maturation (Budoni et al., 2013). The reduction of IgG production was also observed by other authors reporting that both MSC-exosomes and microparticles may increase CD19^+^IL-10^+^ Breg-like population and inhibit plasmablast differentiation by transferring TGF-β, PEG2 and IL1RA (Cosenza et al., 2018). Moreover, MSC-EVs reduce CD27^+^CD19^+^ memory B cell maturation (Balbi et al., 2017). On the other hand, MSC-EVs may sustain, support and enhance the function of human IgG-secreting cells (Nguyen et al., 2018). Notably, MSC-EVs was not capable of significantly affect B cell activation in a strong reactive renal allotransplantation animal model; by contrast, MSC-EVs significantly increased the number of B cells infiltrating the transplanted kidney grafts (Koch et al., 2015). The partial immunomodulation of B cells by MSC soluble factors seems to be preferentially induced by the soluble protein-enriched fraction (PF) rather than by the entire EV-enriched fraction (Carreras-Planella et al., 2019).

#### T Cells

T cells are highly specialized lymphocytes that regulate several aspects of adaptive immunity, such as protection from pathogens, immune surveillance against tumors and alloreaction against non-self-tissues (Kumar et al., 2018). MSCs have a great impact on T cell functions and therefore potentially on the treatment of numerous T-cell mediated reactive conditions (Duffy et al., 2011).

An efficient approach to suppress T cell-mediated immune response is preventing T cell proliferation. Several studies reported that MSC-EVs exert this effect both *in vitro* and *in vivo* in several animal models, such as those reproducing myocardium infarction, experimental allergic asthma and renal IRI (Mokarizadeh et al., 2012; Kilpinen et al., 2013; Blazquez et al., 2014; Romani et al., 2015; Teng et al., 2015; de Castro et al., 2017; Monguió-Tortajada et al., 2017; Cosenza et al., 2018; van den Akker et al., 2018; Ji et al., 2019). The inhibition of T cell proliferation is associated with the reduction or absence of pro-inflammatory cytokines, such as IL-2, IL-6, TNF-α, and IFN-γ (Blazquez et al., 2014; Monguió-Tortajada et al., 2017). Nevertheless, MSC-EVs were capable of increasing T cell number in the graft of a rat renal transplant model for acute rejection associated with the reduction of TNF-α expression and no difference in IL-10 levels (Koch et al., 2015). The inhibition of T cell proliferation by human MSCs is mostly mediated by the upregulation of indoleamine 2,3-dioxygenase (IDO) (Chinnadurai et al., 2015; Wen et al., 2016); however, controversial results are found when T cells are treated with MSC-EVs. Some groups reported no significant changes in IDO expression (Del Fattore et al., 2015; Chen et al., 2016), whereas many authors found high concentrations of IDO inside MSC-EVs (Romani et al., 2015; Zhang et al., 2018b; Serejo et al., 2019). Other groups reported that MSC-EVs have no effect on T cell proliferation, but rather promote T cell apoptosis (Del Fattore et al., 2015; Chen et al., 2016). Conversely, another study reported that MSC-EVs do not alter T cell viability (Monguió-Tortajada et al., 2017). These different findings suggest that a thorough characterization of MSC-EV content and a standardization of the experimental methods are necessary to foresee the biological effects.

Both CD4^+^ and CD8^+^ T cell activation was suppressed by MSC-EVs. At molecular level, the suppression of T cell activation is independent from the antigen presentation due the lack of MHC class I and II as well as other costimulatory molecules on MSC-EV surface (Blazquez et al., 2014; Farinazzo et al., 2018; Dabrowska et al., 2019; Shao et al., 2020). In particular, MSCs constitutively lacking β2-microglobulin, a component of HLA-I involved in CD8^+^ T cell-mediated immune rejection, and the corresponding EVs reduce more efficiently both fibrosis and inflammation in a myocardial infarction animal model compared to the wild-type forms (Shao et al., 2020). The authors reported a greater accumulation of miR-24 in EVs from MSCs constitutively lacking β2-microglobulin, which in turn reduces the expression of the apoptotic protein Bim (Shao et al., 2020). Additionally, MSC-EVs can block CD4^+^ and CD8^+^ T cell differentiation toward effector and memory cells, through a mechanism mediated by TGF-β signaling, respectively (Blazquez et al., 2014; Álvarez et al., 2018).

Modulation of Treg/Th17 and Th1/Th2 balance has been used to explain the regulatory properties of MSC-EVs on T cells. MSC-EVs may promote induction and expansion of Tregsin association with high levels of IL-10 (Mokarizadeh et al., 2012; Kilpinen et al., 2013; Favaro et al., 2014; Del Fattore et al., 2015; Romani et al., 2015; Chen et al., 2016; Nojehdehi et al., 2018; Zhang et al., 2018b; Guo et al., 2019; Ji et al., 2019; Ma et al., 2019), particularly CTLA-4^+^, CD4^+^CD25^+^Foxp3^+^ and Tr1 Treg subpopulations (Chen et al., 2016; Cosenza et al., 2018). Other groups reported no significant changes in Treg number, regardless the higher IL-10 levels after MSC-EV treatment, thus questioning the involvement of Tregs in the upregulation of IL-10 expression by MSC-EVs (Hai et al., 2018). However, the promoting effects of MSC-EVs on Tregs could be partially mediated by their content in TGF-β signaling components (Song et al., 2020). Another possible molecular mechanism is the transfer of miR-1470 from MSC-EVs to CD4^+^ T cells, thus upregulating P27KIP1 expression through c-Jun targeting (Zhuansun et al., 2019). Other miRNAs have been described in this phenomenon, such as miR155-5p, miR-let7b, and miR-let7d. The overexpression in MSC-EVs of miR-155, which targets Sirtuin-1, increases IL-10 and Foxp3 expression in T cells, thus preventing the production of IL-17 and RORC (Zheng et al., 2019). On the other hand, the increase of miR-let7b and miR-let7d may suppress cell proliferation and promote Treg functions, avoiding immune rejection (Wen et al., 2016). Moreover, MSC-EV-mediated proliferation and function of CD4^+^CD25^+^Foxp3^+^ Tregs could involve APC-, but not CD4^+^ T cell-dependent pathways (Du et al., 2018). Regardless the mechanism mainly involved, other *in vivo* models, such as experimental type-1 autoimmune diabetes in T1D mice, clearly showed that the induction of Tregs by MSC-EVs can ameliorate histological signs, thus favoring the regeneration of tissues, i.e., pancreatic islets (Nojehdehi et al., 2018).

Concerning other T-cell subsets, there are only a few works so far. For instance, MSC-EVs may prevent Th17 cell development and IL-17 production (Favaro et al., 2014; Chen et al., 2016; Bai et al., 2017; Shigemoto-Kuroda et al., 2017; Hai et al., 2018; Ji et al., 2019; Ma et al., 2019). MSC-EVs may also inhibit Th17 cell differentiation in ulcerative colitis rat models by increasing histone H3K27me3 methylation and inhibiting its demethylation, thus suggesting that H3K27me3 may be an important target in inflammatory diseases (Chen et al., 2020). Moreover, MSC-EVs can directly prevent Th1 development by promoting Th1 shift toward Th2 cells (Chen et al., 2016; Bai et al., 2017; Shigemoto-Kuroda et al., 2017; Guo et al., 2019) as well as inhibit T follicular helper cells (Hai et al., 2018). Nevertheless, MSC-EVs can also promote autoreactive, IFN-γ-secreting memory Th1 cells by functioning in NOD mice as self-antigen carrier and trigger for autoimmunity (Rahman et al., 2014). In addition, the effect of MSC-EVs on natural killer-T (NK-T) cells has been recently described in a rat model of hepatocellular carcinoma; following EV administration, higher percentages of circulating and intratumoral NK-T cells as well as tumors of smaller size and less aggressive were observed as compared to untreated rats (Ko et al., 2015).

Different mechanisms and factors have been described in the immunomodulatory effect of MSC-EVs toward T-cells. The broad and pleiomorphic activity of MSC-EVs reflects their influence on different signaling pathways of T-cells and microenvironmental cells, such as JAK/STAT or NF-kB (Guo et al., 2019). For instance, MSC-EVs can inhibit T-cell infiltration in the injury site of several diseases as well as the production of several chemokines, such as CCL2, CCL5, CCL21, CXCL1, MIP-1α, MIP-3α, and integrin-dependent chemokines) (Cruz et al., 2015; Bai et al., 2017; Shigemoto-Kuroda et al., 2017; Farinazzo et al., 2018; Hai et al., 2018; Dabrowska et al., 2019) and inflammatory molecules, such as IL-1α, IL-1β, IL-2, IL-5, IL-12, and IL-17 (Favaro et al., 2014; Chen et al., 2016; de Castro et al., 2017; Shigemoto-Kuroda et al., 2017; Hai et al., 2018; Nojehdehi et al., 2018; Dabrowska et al., 2019; Guo et al., 2019; Ji et al., 2019; Ma et al., 2019). By contrast, anti-inflammatory molecules can be induced by MSC-EVs, such as IL-10, TGF-β, and PGE2 (Mokarizadeh et al., 2012; Favaro et al., 2014; Del Fattore et al., 2015; Chen et al., 2016; Nojehdehi et al., 2018; Guo et al., 2019; Ji et al., 2019; Ma et al., 2019). Other factors, such as IL-4, IL-6, IFN-γ, and TNF-α, seem to be variably modulated by MSC-EV (Rahman et al., 2014; de Castro et al., 2017; Shigemoto-Kuroda et al., 2017; Hai et al., 2018; Nojehdehi et al., 2018).

Inflammatory priming may enhance the immunomodulatory properties of MSC-EVs. For instance, inflammatory IL-1β-priming MSC upregulates PD-L1 and TGF-β expression in EVs, leading to a Treg increment in a mouse model of autoimmune encephalomyelitis (Mokarizadeh et al., 2012). A greater accumulation of TGF-β was also reported in IFN-γ-primed MSC-EVs, which also showed low levels of Galectin-1 and IDO, compared to resting MSC-EVs, leading to a suppression of Treg expansion (Serejo et al., 2019). Compared to resting MSC-EVs, TNF-α, and IFN-γ-primed MSC-EVs reduced more the TNF-α and IFN-γ secretion from splenocyte previously activated with lipopolysaccharides and concanavalin A to preferentially stimulate either myeloid cells or T cells, respectively (Harting et al., 2018). According to the authors, the best efficiency of inflammatory priming was probably due to the higher concentration of COX2 and PGE2 in primed MSC-EVs (Harting et al., 2018). Intriguingly, EVs from MSCs pretreated with a combination of anti- and pro-inflammatory cytokines (TGF-β and IFN-γ, respectively) promote Treg expansion more efficiently than MSC-EVs pretreated with TGF-β or IFN-γ only and display higher levels of IDO, IL-10, and IFN-γ (Zhang et al., 2018b). Nevertheless, the promoting effect of inflammatory priming was not confirmed by other authors (Kilpinen et al., 2013; Cosenza et al., 2018), who either found a major effect of resting MSC-EVs, or a negligible effect on T cell proliferation of both resting and primed (TNF-α and IFN-γ) MSC-EVs (Kilpinen et al., 2013; Di Trapani et al., 2016; Cosenza et al., 2018).

Altogether, these data give an idea about the complexity of the interactions and effects that can be mediated by MSC-EVs in physiological and reactive conditions, depending on microenvironmental factors, activating stimuli, effector cell subsets and cellular cross-talk. This scenery becomes even more complex when MSC-EVs are administered as cell-free therapeutic approaches in autoimmune or inflammatory conditions.

### MSC-EV-Based Immunotherapy

MSC systemic administration, which must follow Good Manufacturing Practice (GMP) rules, is not associated to a significant evidence of cell engraftment even in presence of clinical benefit, due to the entrapment of MSCs in the microvasculature of filter organs, such as lungs (Moll et al., 2016; Salvadori et al., 2019). Other biology aspects can interfere with therapeutic efficacy of MSCs. For instance, the quality and the integrity of MSC preparations depends on the isolation, culture, and cryopreservation methods (Moll et al., 2016; Dufrane, 2017; Mastrolia et al., 2019). Although autologous MSCs would be the best choice for MSC therapy, they showed some limitations: patients’ age as well as their genetic traits and medical conditions could reduce the proliferation rate and therapeutic features of MSCs (Pachón-Peña et al., 2016; Dufrane, 2017). Limitations have been also observed in allogeneic MSC transplantation. Indeed, despite MSC have been always considered characterized by a low immunogenic potential, recent studies demonstrated that MSCs may elicit anti-donor immune response (Ankrum et al., 2014; Lohan et al., 2017). Therefore, in order to switch toward a cell-free approach, many groups began to study the immunomodulatory effects of MSC-EVs administered *in vivo*. One of the first clinical setting in which employing MSC-EVs was acute GvHD, the main complication of allogeneic hematopoietic stem cell transplantation (HSCT) (Ferrara et al., 2009; Szyska and Na, 2016; Zeiser and Blazar, 2017). Acute GvHD (aGvHD) occurs within 40 days after HSCT transplantation, as a consequence of interactions between mature donor T cells and host and donor APCs, mounting a strong immune response that eventually lead to host tissue damage (Tyndall and Dazzi, 2008; Zeiser and Blazar, 2017). On the other hand, chronic GvHD (cGvHD) can arise de novo or from aGvHD and is a more complex disease involving not only mature donor T cells, but also auto/alloreactive B cells escaping negative selection (Toubai et al., 2008; Zeiser and Blazar, 2017; Hill et al., 2018). Despite several prophylactic and therapeutic strategies have been developed, the mortality rate of refractory aGvHD is still 70–80%, mostly due to severe secondary infectious complications (Jamil and Mineishi, 2015; Hamilton, 2018).

MSCs initially represented an interesting candidate for cellular therapy to improve HSCT engraftment, prevent graft failure and treat refractory aGvHD. Despite several preclinical and clinical studies showing clinical and survival improvement in MSC-treated patients compared to controls, a significant number of clinical trials failed, especially in adults, probably due to the lack of appropriate knowledge of the mechanisms of action when MSCs are administered *in vivo* (Elgaz et al., 2019; Cheung et al., 2020). For this reason, several groups started to investigate the effectiveness of MSC-EVs in aGvHD *in vivo* models and patients. MSC-EVs may prevent aGvHD onset, attenuate symptoms, and prolong animal survival through several mechanisms. For instance, MSC-EVs is capable of reducing CD8^+^ T cell number, leading to the increase of CD4^+^/CD8^+^ T cell ratio; in addition, they block CD4^+^ T cell migration and activation inside target organs, promote Treg expansion, downregulate IL-2, CCR6, TNF-α, and IFN-γ expression while increasing IL-10, reduce Th17 cell recruitment while lowering RORγτ, STAT3, IL-17, IL-21, IL-22 expression (Wang et al., 2016; Fujii et al., 2018; Lai et al., 2018; Zhang et al., 2018a; Dal Collo et al., 2020). Other potential MSC-EV immunomodulatory mechanisms on T cells involve miR-223 and the adenosine metabolism. miR-223, which is highly expressed in EVs from umbilical cord, is capable of inhibiting allogenic T cell migration and extravasation by targeting ICAM-1, thus leading to a reduction of pro-inflammatory factors and GvHD symptoms (Liu et al., 2020). Regarding adenosine metabolism, it has been observed in a humanized GvHD mouse model that MSC-EVs can transfer CD73 to CD39 enzyme on the surface of tissue-infiltrating Th1 cells, thus inducing a significant production of adenosine that eventually reduces CD39 expression, enhances apoptosis of adenosine A2A receptor-expressing Th1 cells, and downregulates IFN-γ and TNF-α expression, without inducing Tregs (Amarnath et al., 2015). The involvement of adenosine metabolism in T cell modulation was also confirmed by other groups (Kerkelä et al., 2016; Crain et al., 2018). Interestingly, the anti-GvHD function is restricted to MSC-EVs, as human dermal fibroblast-derived EVs are devoid of these effects (Fujii et al., 2018). MSC-EV treatment was also tested in a therapy-refractory GvHD patient, who showed GvHD clinical symptoms improvement and remained stable for several months (Kordelas et al., 2014): MSC-EV preparations contained high concentrations of IL-10, TGF-β, and HLA-G that paralleled with the decrease in the number of both PBMCs releasing IL-1β, TNF-α, and IFN-γ and stimulated NK cells releasing TNFα- or IFN-γ (Kordelas et al., 2014).

Unfortunately, not all EV preparations from MSCs are functionally equivalent (Madel et al., 2019). Therefore, it is necessary to characterize the functional activity of MSC-EV preparations and to identify predictive tests that may foresee the clinical benefit. Kordelas et al. (2019) proposed an *in vitro* assay to monitor the impact of different EV preparations from human donor bone marrow MSCs (BM-MSCs)-MSCs on T cell differentiation and corresponding cytokine production. Recently, a functional *in vitro* assay was suggested to assess the MSC-EV therapeutic dose (EV-TD) *in vivo* in a mouse model of aGvHD; EV-TD, associated with the improvement of mouse overall survival, corresponded to 10-fold the EV immunomodulatory functional unit (EV-IFU), i.e. the lowest concentration *in vitro* of resting MSC-EV-pool leading to at least threefold increase of Tregs compared to control (Dal Collo et al., 2020). Nevertheless, all these assays need to be validated in a large cohort of patients before being accepted as predictive methods of MSC-EV therapeutic efficacy.

Other clinical studies employing MSC-EVs as treatment of many diseases with inflammatory phenomena are reported on clinicaltrials.gov. According to our search, using the terms “mesenchymal extracellular vesicles” and “stromal extracellular vesicles,” only three clinical studies have been registered concerning bronchopulmonary dysplasia (NCT03857841)^1^, osteoarthritis (NCT04223622)^2^, and dystrophic epidermolysis bullosa (NCT04173650)^3^. In particular, NCT03857841 study will employ UNEX-42, a preparation of EVs secreted from human BM-MSCs suspended in phosphate-buffered saline; NCT04223622 study will use the entire secretome or EVs derived from adipogenic MSCs; and NCT04173650 study will employ AGLE-102, an allogeneic derived EV product derived from normal donor MSCs. However, all studies are currently ongoing and no clear-cut results have been reported so far.

## Conclusion

Immunomodulatory capacity of MSCs is associated, at least in part, with the release of EVs. The ability of MSC-EVs to affect immune response, promoting immunotolerance in tissue microenvironment, opens new cues on intercellular communication through soluble factors and makes MSC-EVs a new promising therapeutic strategy for the treatment of many inflammatory disorders. Compared to cell therapy, EV treatment offers a number of advantages in terms of higher distribution in target organs, lower immunogenicity and tumorigenicity as well as easier handling and preparation procedures. Unfortunately, MSC-EVs can have variable biological effects on the same effector cell type depending on different factors, such as the quality of primary cells, MSC source, culture conditions, preconditioning with inflammatory cytokines, cryopreservation methods, purification and quantification protocols, etc. (Théry et al., 2018). These premises, together with the lack of standardized approaches, specific dosing and defined quality controls for clinical use, require further investigations before transferring EV-based treatments from bench to bedside.

## Author Contributions

RB wrote the manuscript and prepared the figure. PT and IT critically revised the manuscript. MK planned and revised the final version of the manuscript. All authors approved the submitted version of the manuscript.

## Conflict of Interest

The authors declare that the research was conducted in the absence of any commercial or financial relationships that could be construed as a potential conflict of interest.

## Footnotes

1 https://clinicaltrials.gov/ct2/show/NCT03857841

2 https://clinicaltrials.gov/ct2/show/study/NCT04223622

3 https://clinicaltrials.gov/ct2/show/study/NCT04173650
